# Editorial: Avian Models for Social Cohesion

**DOI:** 10.3389/fphys.2019.01533

**Published:** 2020-01-08

**Authors:** Andras Csillag, Giorgio Vallortigara, Gergely Zachar

**Affiliations:** ^1^Department of Anatomy, Histology and Embryology, Faculty of Medicine, Semmelweis University, Budapest, Hungary; ^2^Center for Mind and Brain Sciences, University of Trento, Rovereto, Italy

**Keywords:** bird, affiliation, imprinting, social perception, flocking, ASD, forebrain evolution

As a cover term, social cohesion comprises different levels of explanation for a fundamental feature of numerous vertebrate species: the ability to form groups. Birds have long been considered good examples for sociability, with iconic capabilites such as birdsong, migration, or diverse mating systems and parental behavior. Apart from the fascination evoked by the apparent complexity of avian affiliatory behaviors, an impressive number of studies have been focusing on the underlying mechanisms. It is becoming increasingly evident that the validity of molecular, neurophysiological, and neuroanatomical correlates of avian social behaviors extends far beyond the realm of birds. More recent research has further corroborated the value of avian models for normal or impaired social cohesion, even for certain social deficits occurring in humans.

The present selection of articles represents molecular, behavioral, evolutionary, and theoretical approaches of investigation. According to their focus, the articles can be assigned to the following main categories: (i) Imprinting as one fundamental tool in early bond formation; (ii) Predispositions and socially relevant perception mechanisms; (iii) Adaptive processes underlying flocking; (iv) Evolutionary and developmental trends determining sociability in vertebrates. In this editorial summary we, as Guest Editors, attempt to provide a progress report in those fields, illuminating The Research Topic for the Reader by bringing them, if momentarily, into the limelight.

*Ad (i)* By revisiting visual imprinting, the review by McCabe discusses this process in the framework of filial bond forming and, in more general terms, social recognition. It also gives a promising account of new perspectives in the investigation of physiological mechanisms underlying imprinting. One such recently discovered factor determining the sensitive period for imprinting is triiodothyronine (T3). The study by Aoki et al. further elucidates the molecular basis of this T3 dependent regulatory mechanism by demonstrating an increase in GABA-A receptor expression and a contrasting decrease in GABA-B receptor expression in the early post-hatch period. The authors concluded that GABA-B receptors facilitate imprinting downstream to T3, whereas GABA-A receptors contribute to termination of the imprintable period. The paper by Miura et al. further elaborates on the role of T3 by showing that, as one possible mechanism to prolong the sensitive period for imprinting, T3 also promotes the manifestation of predisposed preference for biological motion (BM) in 1-day-old (but not in 4-day-old) chicks. Partial re-sensitization of BM preference and filial imprinting with T3 corroborate its role in the flexibility of the imprintable period. Another aspect of imprinting concerns the association with memory formation for the imprinting stimulus (with relevance to social recognition). The study by Tiunova et al. demonstrated an elevation of c-fos induction in the hippocampus on first presentation of the imprinting stimulus, but not after memory retrieval, while in the IMM, mediorostral nidopallium/mesopallium and hyperpallium densocellulare, c-fos activation was induced by retrieval of only the remote but not of recent memory. The results cast new light on a perplexing problem of an apparent “migration” of the socially relevant memory trace from acquisition to consolidation.

*Ad (ii)*. A key element of social recognition involves categorical representation of biologically relevant percepts such as facial features. The study by Clark et al. was based on electrophysiological recordings from four relevant forebrain structures associated with the tectofugal visual system (entopallium, mesopallium ventrolaterale, nidopallium frontolaterale, area temporo-parieto-occipitalis) in a discrimination task of pigeons. No “face-selective” neurons were detected in any of the regions, suggesting a predisposition of birds for a more global combination of features, also subserving perception of faces. Early predispositions are implicated in the recognition of conspecifics, social partners, or predators. Similar processes likely occur in human newborn infants, and are potentially impaired in newborns at risk of Autism Spectrum Disorder (ASD). Using a domestic chick model, Lorenzi et al. found that, similarly to impairments of preference for static social stimuli, valproic acid (VPA) exposure *in ovo* specifically affects also the preference for animate motion stimuli. The study underlines the importance of predispositions in the development and early diagnosis of ASD in human neonates. The study by Zachar et al. focuses on another aspect of VPA-related effects. While the preference for large vs. small groups of conspecifics, and for partners with intact facial features over those with blurred faces, as well as early adaptive learning, were unaffected by VPA, social exploration and the recognition of familiar conspecifics were attenuated after VPA treatment. The findings suggest an importance of early social exploration in the development of ASD.

*Ad (iii)*. How and why birds prefer their conspecifics is reviewed by Riters et al. with a comprehensive overview of group forming behaviors. Flocking offers distinct adaptive benefits for gregarious species of birds without an immediate reward (survival, food reward, mating) for the individual. Thus, in the long run, such behavior has to be promoted by reward (positive social interactions that “feel good”) and by reinforcement (reducing the negative affective state due to social isolation). Both modalities can be controlled by the mu opioid receptors in the medial preoptic area, connected with the periaqueductal gray and ventral tegmental area. Based on experimental evidence from starlings (*Sturnus vulgaris*), the paper reviews and extends current knowledge on the motivation to form non-sexual social groups.

*Ad (iv)*. Sociability necessitates an interplay between different neural systems. The large-scale review by Medina et al. summarizes the phylogenetic processes leading to divergent yet comparable neuroanatomical systems in birds and mammals. The account concentrates on two interrelated brain networks instrumental in sociability: one including the pallial (basolateral) amygdala, temporal and temporoparietal neocortices, and orbitofrontal cortex, involved in social perception and decision-making, and another comprising the medial extended amygdala, ventromedial striatum (nucl. accumbens), and ventromedial hypothalamus, related to affiliation. The study gives a detailed comparison of available neuroanatomical, evolutionary and developmental data between the relevant neural systems of mammals and their sauropsid equivalents.

## Author Contributions

All authors listed have made a substantial, direct and intellectual contribution to the work, and approved it for publication.

### Conflict of Interest

The authors declare that the research was conducted in the absence of any commercial or financial relationships that could be construed as a potential conflict of interest.

